# High Infection-Related Mortality in Pediatric Acute Myeloid Leukemia without Preventive Antibiotics and Antifungals: Retrospective Cohort Study of a Single Center from a Middle-Income Country

**DOI:** 10.4274/tjh.2017.0052

**Published:** 2017-12-01

**Authors:** Emine Zengin, Nazan Sarper, Sema Aylan Gelen, Uğur Demirsoy, Meriban Karadoğan, Suar Çakı Kılıç, Selim Öncel, Emin Sami Arısoy, Devrim Dündar

**Affiliations:** 1 Kocaeli University Faculty of Medicine, Department of Pediatrics, Division of Pediatric Hematology, Kocaeli, Turkey; 2 Kocaeli University Faculty of Medicine, Department of Pediatrics, Division of Pediatric Infectious Diseases, Kocaeli, Turkey; 3 Kocaeli University Faculty of Medicine, Department of Microbiology, Kocaeli, Turkey

**Keywords:** Acute myeloid leukemia, Pediatric leukemia, Febrile neutropenia, Infection

## Abstract

**Objective:**

This study aimed to evaluate infection-related mortality in patients with acute myeloid leukemia (AML) treated without preventive antibiotics and antifungals in a middle-income country.

**Materials and Methods:**

Infection-related mortality was evaluated retrospectively in 49 pediatric patients.

**Results:**

A total of 173 chemotherapy courses were administered as first-line chemotherapy. Four patients died during induction: one patient due to intracranial bleeding, two patients due to typhlitis, and one patient due to invasive fungal infection with pulmonary vascular invasion and massive bleeding. Another two patients died with resistant disease. During consolidation there were four infection-related deaths and one death due to cardiotoxicity. In first-line chemotherapy mortality was 22% (11/49); infection-related mortality was 14% (7/49). Event-free survival and overall survival at 6 years were 42.9% and 61.2% (95% CI: 44-76 and 66-99 months), respectively.

**Conclusion:**

Due to considerable infection-related deaths, antibacterial and mold-active antifungal prophylaxis may be tried during neutropenic periods in pediatric AML.

## Abstract (tr)

**Amaç:**

Orta gelir düzeyindeki bir ülkede koruyucu antibiyotik ve antifungal kullanılmadan tedavi edilen akut miyeloid lösemi (AML) tanılı hastalarda enfeksiyona bağlı ölümlerin değerlendirilmesidir.

**Gereç ve Yöntemler:**

Kırk dokuz AML tanılı çocuk hastada enfeksiyona bağlı ölümler retrospektif olarak değerlendirildi.

**Bulgular:**

İlk basamak tedavi olarak toplam 173 kemoterapi kürü uygulandı. İndüksiyon sırasında bir hasta beyin kanaması, iki hasta tiflit ve bir hasta invazif mantar enfeksiyonunun akciğer damar duvarını hasarlaması sonucu gelişen yoğun kanamayla olmak üzere toplam dört hasta kaybedildi. İki hasta da dirençli hastalık ile kaybedildi. Konsolidasyon sırasında beş ölüm vardı; dördü bakteriyemi ve sepsis, biri kardiyotoksisite ilişkiliydi. İlk basamak tedavide ölüm oranı %22 (11/49) olup, bunların %14’ü (7/49) enfeksiyonla ilişkiliydi. Olaysız sağkalım ve genel sağkalım sırasıyla 6 yılda %42,9 ve %61,2 (%95 güven aralığı: 44-76 ve 66-99 ay) bulundu.

**Sonuç:**

Dikkate değer sayıda enfeksiyon ölümleri nedeniyle antibakteriyel ve küf mantarlarına etkili koruyucu tedaviler, orta-gelir düzeyindeki ülkelerde, AML tanılı çocuk hastaların nötropenik dönemlerinde kullanılabilir.

## INTRODUCTION

Children and adolescents with acute myeloid leukemia (AML) are at risk of severe infectious complications as a result of prolonged neutropenia. It was reported that the main causes of death during chemotherapy are infections [1]. Studies show that prophylaxis with antibiotics and antifungals reduced infections, hospitalization days, and mortality [2,3,4], but the emergence of resistance, particularly in vancomycin-resistant enterococci (VRE) and gram-negative bacteria, is another dilemma [5].

The aim of this study was to document infection-related mortality (IRM) of patients with de novo AML and myelodysplastic syndrome (MDS)/AML during first-line chemotherapy courses and compare the results with the literature data.

## MATERIALS AND METHODS

This retrospective study was performed in a university hospital’s pediatric hematology unit. The hospital records of all de novo AML/MDS patients aged <18 years diagnosed from June 2005 through February 2016 were reviewed by two experienced hematologists of the unit. Patients with Down syndrome were also included. Before starting chemotherapy, all the parents/legal guardians gave informed consent for the treatment and for the usage of patient data in the research. The ethics committee of the institution also approved the study.

The United Kingdom Medical Research Council (MRC) AML-10 chemotherapy protocol was used as first-line chemotherapy with some modifications and no randomizations [1]. A modification was the substitution of amsacrine with idarubicine 10 mg/m^2^ on days 0 and 1 in some patients due to unavailability of the drug. Patients with Down syndrome were treated with reduced-intensity chemotherapy. Patients stayed in two-bed rooms with a bathroom and there was no high-efficiency particulate air (HEPA) filtration. Co-trimoxazole prophylaxis was administered. Granulocyte colony stimulating factor (G-CSF) was only used in consolidation phases if there was severe infection with hemodynamic instability. Patients generally were not discharged until remission, but in the subsequent chemotherapy courses they were followed as outpatients if there were no infections.

In the first years of the study, ceftazidime and then piperacillin/tazobactam or cefepime were used as empirical monotherapy. Carbapenems and teicoplanin were administered as initial empirical therapy in hemodynamically unstable patients. If there were any respiratory symptoms at initial presentation of febrile neutropenia or if fever persisted longer than 96 h, serum galactomannan monitoring was started and chest computerized tomography and abdominal ultrasound imaging were performed. Empirical mold-active antifungals were introduced after 96 h. Patients were referred to some other centers when hematopoietic stem cell transplantation (HSCT) was indicated.

### Statistical Analysis

Data were analyzed using SPSS 13. Descriptive statistics were employed and are reported as absolute frequencies or percentages for qualitative data and as medians and range or means and standard deviations for quantitative data. Comparisons of frequency distribution were analyzed with the nonparametric statistics of the chi-square test or the Kruskal-Wallis test. For survival, Kaplan-Meier analysis was performed. All tests were two-tailed and p<0.05 was considered statistically significant.

## RESULTS

**Figure 1:**
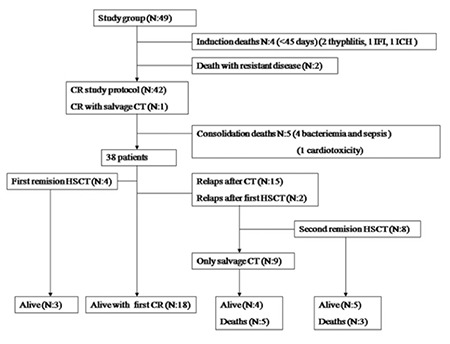
The distribution of first-line treatment modalities and outcomes. 2-CdA: Cladribine, HCL: hairy cell leukemia, INF-α: interferon-alpha, RTX: rituximab, SPL: splenectomy, ORR: overall response rate, NRR: non-response rate. *See text for details.

Forty-nine patients (32 boys, 17 girls) with AML were diagnosed and 173 chemotherapy courses were administered as first-line chemotherapy. Ten of the patients had acute promyelocytic leukemia, 4 patients had Down syndrome, and 3 patients had MDS/AML (one had myelofibrosis). The overall remission rate with first-line chemotherapy was 85.7%. During induction and consolidation there were 11 deaths. In first-line chemotherapy mortality was 22% (11/49); IRM was 14% (7/49) (Figure 1).

In 682 sterile-site cultures, 47 cases of pathogen growth were observed. The isolated pathogens of the three infection-related deaths were Candida guilliermondii (septicemia and pneumonia), extended spectrum β-lactamase-positive Klebsiella pneumoniae (septicemia), and Enterobacter cloacae (septicemia). Except for the patient with Candida septicemia who had sudden pulmonary hemorrhage, all the patients required intensive care. Gram-negative bacteria isolation was more frequent than gram-positive (67.2% versus 32.7%). Escherichia coli and K. pneumoniae were the most predominant isolates. Viridans streptococci were rarely isolated; there were three Streptococcus mitis isolations from this group. Characteristics of the infectious episodes and identified pathogens are demonstrated in Tables 1 and 2, respectively.

In the first remission, 4 matched related donor (MRD) and 1 matched unrelated donor (MUD) transplants were performed. Eight patients had allogeneic HSCT in the second remission (4 MRD, 2 MUD, and 2 haploidentical transplants), and two of these patients had second transplants. Of the 11 transplanted patients, 8 were alive in a median of 54 (range: 6-84) months (Figure 1).

**Figure 2:**
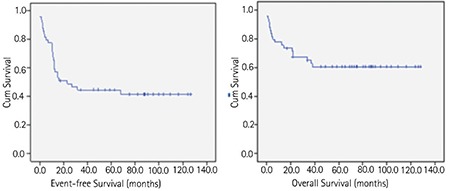
Kaplan-Meier analysis of event-free and overall survival.

Event-free survival and overall survival at 6 years were 42.9% and 61.2% (95% CI: 44-76 and 66-99 months), respectively (Figure 2).

## DISCUSSION

Sixty-one percent of the patients were alive during the retrospective study period. During the first-line treatment there were 11 deaths (22%), 7 of which (14%) were infection-related. In the present study, with less intensive chemotherapy, out of 4 patients with Down syndrome there was only one infection-related death during induction.

In a multicenter study of children also treated with the MRCAML-10 protocol, IRM was 9.1% [1]. Single-occupancy rooms with HEPA filtration and more training about hand washing may reduce infections.

It was reported that prophylactic oral cephalosporins did not significantly reduce bacterial sepsis, but intravenous cefepime or vancomycin with oral ciprofloxacin reduced bacterial sepsis and days of hospitalization. Prophylactic oral voriconazole was also used in that study but it did not reduce fungal infections. IRM was 2.5% [2].

In a multicenter trial some centers used antibacterial (penicillin or vancomycin and others), antifungal, and G-CSF prophylaxis. All the centers used fluconazole prophylaxis. The authors concluded that antibacterial prophylaxis reduced sterile-site infections. Prophylactic G-CSF reduced bacterial and Clostridium difficile infections. Mandatory hospitalization did not reduce bacterial/fungal infection and nonrelapse mortality but did increase C. difficile infections [6].

We hospitalized patients until remission but in the subsequent courses we discharged them. Mandatory hospitalization might prevent some infection-related deaths. One patient died at home with infection and another’s admission was delayed. In another study, vancomycin and ceftazidime or cefepime prophylaxis was administered in addition to fluconazole, voriconazole, or micafungin. There was only one infection-related death due to candidal pneumonia. When induction I was excluded, preventive antibiotics reduced infections compared to no prophylaxis. The hospital rooms were all single-occupancies and most of them had HEPA filtration. However, after emergence of VRE and C. difficile infections, vancomycin was removed from the preventive therapy and cefepime mono therapy was started [7].

In the multicenter trial AML-Berlin-Frankfurt-Münster (BFM) 2004, excluding patients with Down syndrome, the infection-related morbidity rate was 3.3 per patient and IRM was only 1.5%. There was a reduction in IRM compared to the previous AML-BFM 93 study. This was explained by the administration of anti-mold active prophylaxis (amphotericin B, voriconazole, or posaconazole) in more than 70% of the chemotherapy cycles and prompt institution of empiric therapy that should include a glycopeptide in severely ill patients and regular training courses in the education of the pediatric hematologists [8].

## CONCLUSION

In a multicenter study of pediatric AML from Turkey, similar to our study, overall survival was 58.8% and IRM was 16.6%. The authors also suggested that better supportive care may improve outcomes [9].

In light of the above literature we think that, to reduce IRM, we should try prophylaxis in patients with pediatric AML. Prophylactic piperacillin/tazobactam or cefepime and voriconazole can be administered in the induction. After induction oral ciprofloxacin and voriconazole can be tried in preventive therapy when the absolute neutrophil count (ANC) falls below 0.5x10^9^/L and discontinued once the ANC recovers to >0.1x10^9^/L. Prophylaxis may justify the potential risk from emerging resistant bacteria.

## Figures and Tables

**Table 1:**
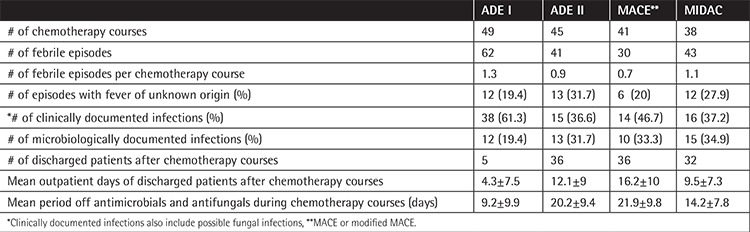
Characteristics of the infectious episodes in pediatric patients with acute myeloid leukemia.

**Table 2:**
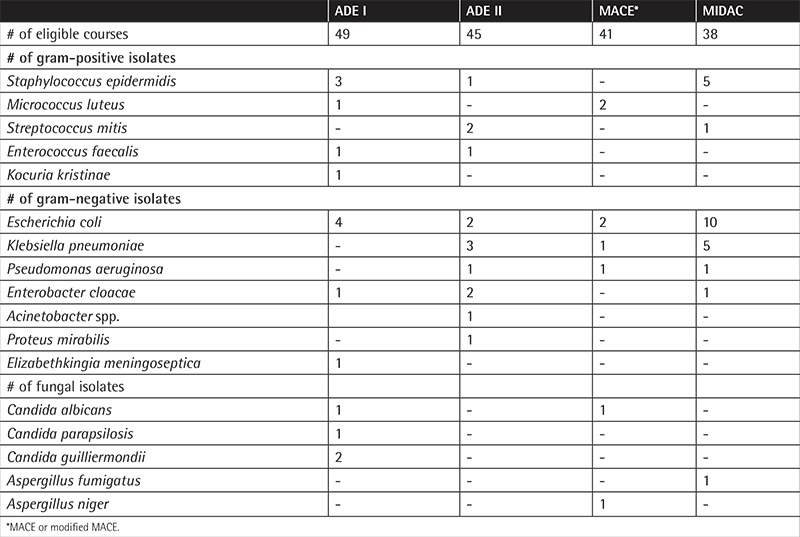
Isolated pathogens during chemotherapy courses of pediatric patients with acute myeloblastic leukemia.
